# Effect of crop plants on fitness costs associated with resistance to *Bacillus thuringiensis* toxins Cry1Ac and Cry2Ab in cabbage loopers

**DOI:** 10.1038/srep20959

**Published:** 2016-02-12

**Authors:** Ran Wang, Guillaume Tetreau, Ping Wang

**Affiliations:** 1 Department of Entomology, Cornell University, New York State Agricultural Experiment Station, Geneva, NY 14456, USA; 2 Department of Entomology, Nanjing Agricultural University, Nanjing 210095, China

## Abstract

Fitness costs associated with resistance to *Bacillus thuringiensis* (Bt) toxins critically impact the development of resistance in insect populations. In this study, the fitness costs in *Trichoplusia ni* strains associated with two genetically independent resistance mechanisms to Bt toxins Cry1Ac and Cry2Ab, individually and in combination, on four crop plants (cabbage, cotton, tobacco and tomato) were analyzed, in comparison with their near-isogenic susceptible strain. The net reproductive rate (*R*_*0*_) and intrinsic rate of increase (*r*) of the *T. ni* strains, regardless of their resistance traits, were strongly affected by the host plants. The *ABCC2* gene-linked mechanism of Cry1Ac resistance was associated with relatively low fitness costs, while the Cry2Ab resistance mechanism was associated with higher fitness costs. The fitness costs in the presence of both resistance mechanisms in *T. ni* appeared to be non-additive. The relative fitness of Bt-resistant *T. ni* depended on the specific resistance mechanisms as well as host plants. In addition to difference in survivorship and fecundity, an asynchrony of adult emergence was observed among *T. ni* with different resistance mechanisms and on different host plants. Therefore, mechanisms of resistance and host plants available in the field are both important factors affecting development of Bt resistance in insects.

Transgenic crops expressing the environmentally benign insecticidal toxins from *Bacillus thuringiensis* (Bt) are now widely planted in over 70 million hectares worldwide for pest control^1^. However, the benefits of Bt-crops can be diminished if insects develop resistance to the Bt toxins. Bt resistance has been well documented in a number of insect pests under laboratory selections and in the field^2^. It is known that insect resistance to Bt toxins is often associated with fitness costs, the reduction of fitness relative to susceptible individuals, which lead to reduced competitiveness of resistant insects on non-Bt host plants and consequently critically impact the selection of Bt resistant alleles in insect populations^3^. Therefore, with the increasing application of Bt toxins in transgenic crops, studies on Bt resistance-associated fitness costs have been increasingly reported since the 1990s.

Bt resistance associated fitness costs have been studied on artificial diet in several lepidopteran pests, including *Helicoverpa armigera*^4^, *Heliothis virescens*^5^, *Ostrinia nubilalis*^6^, *Pectinophora gossypiella*^7^, *Plutella xylostella*^8^, *Spodoptera exigua*^9^ and *Trichoplusia ni*^10^, or on their primary host plants cabbage for *P. xylostella*^8^,^11^,^12^,^13^, tomato, cucumber and pepper plants for *T. ni*^14^, cotton plants for *H. armigera*^15^, *H. virescens*^16^ and *P. gossypiella*^17^, maize for *Busseola fusca*^18^, *O. nubilalis*^19^, *H. zea*^20^ and *S. frugiperda*^21^ and tobacco for *S. litura*^22^. The fitness costs can be affected by the genetic backgrounds of the insects, mechanisms of resistance, host plants, and other dietary and ecological factors^7^,^11^,^12^,^13^,^14^, which complicate the fitness cost analysis and could lead to spurious data interpretations^3^. Fitness costs associated with Bt resistance in insects often become higher when food quality decreases, e.g. feeding alternative plants^3^,^11^. In the field, insects feed on various host plants, depending on the host ranges of the insects and field situations, and most agriculturally important lepidopteran pests are polyphagous herbivores feeding on a range of host plants. Different host plants vary in their nutritional quality for insects and also vary in their insecticidal secondary metabolites that impose chemical stress on the insects, both of which could affect the fitness costs associated with insect resistance^11^,^12^,^14^. Therefore, to understand the fitness costs associated with Bt resistance, it is important to dissect the fitness costs associated with specific resistance mechanisms on specific host plants in insect populations in the same genetic background.

The cabbage looper, *T. ni*, is a highly polyphagous insect with an exceptionally broad and diverse range of host plants, including over 160 plant species in 36 families^23^. In *T. ni*, more than 70 cases of resistance to 14 different insecticides have been documented (http://www.pesticideresistance.org). More notably, *T. ni* is one of the only two insect pests that have developed resistance to *Bt* formulations in agricultural settings^24^. From a greenhouse-evolved Bt-resistant *T. ni* population, two genetically independent Bt resistance traits, Cry1Ac resistance and Cry2Ab resistance, have been isolated and introgressed into a highly homozygous susceptible laboratory *T. ni* strain^25^,^26^. The Cry1Ac resistance in *T. ni* has been identified to be conferred by the same major genetic mechanism for Cry1Ac resistance shared by several lepidopteran pests^27^,^28^,^29^, enabling *T. ni* to survive on Bt-cotton plants expressing the Cry1Ac toxin^30^. Combining the resistance mechanisms to Cry1Ac and Cry2Ab in *T. ni* allows the insects to survive on the widely planted major pyramided dual-toxin transgenic Bt-cotton plants (Bollgard II)^30^. The broad host range of *T. ni* makes this insect a pest of numerous important agricultural crops, from cruciferous vegetables to the field crop cotton^23^, but also provides an ideal system to study the effects of crop plants with different nutritional quality and different secondary metabolites on the fitness costs associated with specific Bt resistance mechanisms. In this study, we used the unique near-isogenic *T. ni* strains^25^,^26^,^30^,^31^ as a biological system to dissect the fitness costs associated with the mechanisms of Cry1Ac resistance, Cry2Ab resistance and a combination of both mechanisms of resistance to Cry1Ac and Cry2Ab in *T. ni*. on artificial diet, on their preferred host plant (cabbage) and on alternative host plants (cotton, tobacco and tomato).

## Results

### Population growth of *T. ni* is affected by the mechanisms of resistance to Bt and host plants

Both the Bt-susceptible Cornell strain^30^ and three near-isogenic *T. ni* strains resistant to Cry1Ac, strain GLEN-Cry1Ac-BCS^25^, to Cry2Ab, strain GLEN-Cry2Ab-BCS^26^, and to both Cry1Ac and Cry2Ab, strain GLEN-Cry1Ac+Cry2Ab-BCS^30^, all survived and completed their life cycles on the foliage of four host plants (cabbage, cotton, tobacco and tomato) tested. The net reproductive rate (NRR) (*R*_*0*_) for each strain was far greater than the critical value 1 (ranging from 31.0 to 347.9) on the four host plants. However, the NRRs were affected by both host plants and the Cry toxin resistance traits (Table 1). Compared to feeding on artificial diet as control, feeding on plant foliage reduced the population growth, showing a decrease of the *R*_*0*_ values from 252.3–347.9 to 31.0–98.7. On cabbage, all strains showed a similar NRR reduction to 32–35% of that on artificial diet. The *T. ni* strains resistant to Cry2Ab, GLEN-Cry2Ab-BCS and GLEN-Cry1Ac+Cry2Ab-BCS, showed a greater decrease of *R*_*0*_ (decreased to 11–18% and 13–18%, respectively for the two strains) on cotton, tobacco and tomato leaves than the Cry1Ac-resistant strain GLEN-Cry1Ac-BCS (decreased to 18–22%) and the Cornell strain (decreased to 26–35%). The decrease of fitness of *T. ni* associated with the resistance was relatively low on artificial diet (relative fitness *w* = 0.72–0.80) and cabbage (*w* = 0.78–0.89) but was high on cotton (*w* = 0.36–0.54), tobacco (*w* = 0.46–0.78) and tomato (*w* = 0.25–0.50) leaves (Table 1). The fitness cost measured by *w* values associated with Cry2Ab resistance was greater than that with Cry1Ac resistance on the three plants (Table 1).

Analysis of population growth of the *T. ni* strains using the intrinsic rate of increase (IRI) (*r*) (Table 2) showed similar patterns as those from the analysis based on NRR described above (Table 1). The *r* values for the *T. ni* strains on four plants were all >0, ranging from 0.116 to 0.297, indicating that the *T. ni* populations could grow on the foliage of the four plants. *T. ni* strains showed a higher reduction of *r* values on the foliage of cotton, tobacco and tomato plants than on cabbage and artificial diet, and the fitness cost associated with Cry2Ab resistance was greater than that with Cry1Ac resistance (Table 2).

### Fecundity of *T. ni* is affected by the mechanisms of resistance and host plants

A generalized linear model analysis indicated that females of *T. ni* fed on plant foliage laid significantly less eggs than those on artificial diet (“plant effect”, *df* = 4, *p* < 2.2 × 10^−16^) (Table 3, Fig. S1). Feeding on cabbage had the smallest negative effect comparing to feeding on the plants tested, causing 17–22% reduction of the total number of eggs as compared to the control feeding artificial diet; while feeding on tomato foliage had the greatest effect, causing a 38–54% reduction of egg numbers. The reduction of egg laying associated with the resistance to Cry1Ac and Cry2Ab in *T. ni* was minimal on artificial diet (reduction of 5–8%) and cabbage (reduction of 3–8%). However, feeding the other three plants resulted in a 11–17%, 13–26% and 20–30% reduction of egg laying, respectively, in the strains GLEN-Cry1Ac-BCS, GLEN-Cry2Ab-BCS and GLEN-Cry1Ac + Cry2Ab BCS (“strain effect”, *df* = 3, *p* < 2.2 × 10^−16^) (Table 3, Fig. S1). The greatest reduction of egg laying observed was in the GLEN-Cry2Ab-BCS strain on tomato foliage, which also showed the most notable effect of interaction of the two factors (“plant × strain”, *df* = 12, *p* < 2.2 × 10^−16^) (Fig. S1).

**Figure 1:**
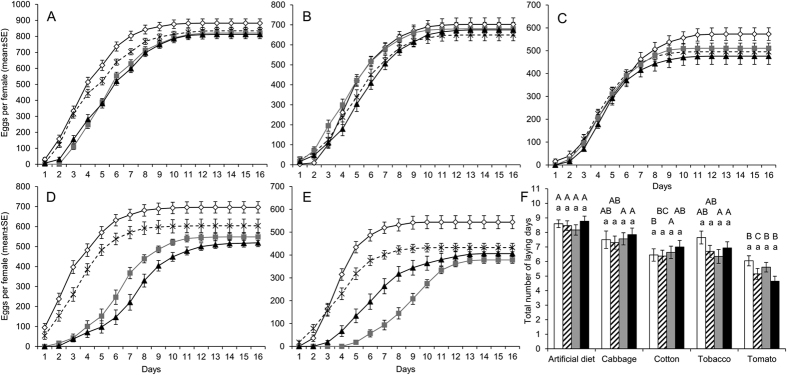
Kinetics of egg laying by females from the susceptible (white diamond, black line), Cry1Ac (cross, dotted line), Cry2Ab (grey square, grey line) and Cry1Ac-Cry2Ab resistant strains (black triangle, black line) reared on artificial diet (**A**), cabbage (**B**), cotton (**C**), tobacco (**D**) or tomato (**E**). (**F**). Total number of egg laying days for the susceptible (white), Cry1Ac (striped), Cry2Ab (grey) and Cry1Ac-Cry2Ab resistant strains (black) reared on each plant. The observations from different *T. ni* strains on the same plant that were not statistically different from each other are indicated by the same letter in lower case above the bars. The observations from the same *T. ni* strain on different plants that were not statistically different from each other are indicated by the same letter in upper case above the bars. Values are indicated as mean ± SE.

The kinetics of egg laying were significantly affected by host plants (“plant effect”, *df* = 4, *p* < 2.2 × 10^−16^) and by the resistance traits (“strain effect”, *df* = 3, *p* < 2.2 × 10^−16^) (Fig. 1, Fig. S1). No significant difference in egg laying dynamics between the four strains was observed when the *T. ni* were grown on artificial diet, and on cabbage and cotton foliage (Fig. 1) (two-sample two-sided Kolmogorov-Smirnov analysis, *p* > 0.05). However, a significant difference was observed in Cry2Ab-resistant *T. ni* on tobacco (D = 9.37%, *p* = 0.015 for GLEN-Cry2Ab-BCS; D = 8.33%, *p* = 0.036 for GLEN-Cry1Ac+Cry2Ab-BCS) and on tomato (D = 8.13%, *p* = 0.042 for GLEN-Cry2Ab-BCS; D = 8.96%, *p* = 0.021 for GLEN-Cry1Ac+Cry2Ab-BCS). This is consistent with the statistically significant interaction between host plants and resistance traits by GLM analysis (“plant x strain”, *df* = 11, *p* < 2.2 10^−16^) (Table 3, Fig. S1). The total number of egg laying days was also significantly affected by the host plants (“plant effect”, *df* = 4, *p* = 2.3 10^−14^), showing a decrease of 2.6–4.1 days of egg laying days on tomato for all strains regardless of resistant trait (“strain effect”, *df* = 3, *p* = 0.61) (Fig. 1F, Fig. S1). A decrease in egg hatching rate was observed in all resistant strains (“strain effect”, *df* = 3, *p* < 2.2 10^−16^) (Table 3, Fig. S1). However, the level of decrease in hatchability was very minimal, with the largest reduction to be only 6.2% to 8.9% observed in eggs from the GLEN-Cry1Ac+Cry2Ab-BCS strain on cabbage and cotton plants (“plant x strain”, *df* = 12, *p* = 1.6 10^−03^) (Table 3, Fig. S1).

### Development of *T. ni* is affected by the mechanisms of resistance and host plants

The duration of larval development was affected by *T. ni* strains (“strain effect”, *df* = 3, *p* < 2.2 10^−16^) and host plants (“plant effect”, *df* = 4, *p* = 5.1 10^−04^) without statistically significant interaction of the two factors (“plant x strain”, *df* = 12, *p* = 0.27) (Fig. S1, Table 4). The larval stage was 2.4 − 3.2, 8.3 −12.9, 8.1 − 9.9 and 6.5 − 8.8 days longer on cabbage, cotton, tobacco and tomato foliage, respectively, than that on artificial diet (Table 4). On artificial diet, the pupal stage was 8 days, regardless of the *T. ni* strains (“strain effect”, *df* = 3, *p* = 0.23) (Table 4). The duration of pupal stage varied within 1 day among the treatments (“plant effect”, *df* = 4, *p* = 1.0 10^−10^), with the only exception for the Cry2Ab resistant strains on cotton (the pupal stage was 3 days shorter than susceptible strain) (“plant x strain”, *df* = 12, *p* = 9.2 10^−05^) (Table 4, Fig. S1).

The gender had no significant effect on pupal weight (“sex effect”, *df* = 1, *p* = 0.84) (Fig. S1). Pupae from cabbage showed no difference in weight from those on artificial diet, while those from cotton, tobacco and tomato plants showed a 55–70% decrease in pupal weight (“plant effect”, *df* = 4, *p* = 5.2 10^−02^) (Table 5, Fig. S1). Similarly, the rate of adult emergence from pupae was affected by the host plants (“plant effect”, *df* = 4, *p* = 2.6 10^−03^) (Fig. S1), with the most significant reduction of emergence observed on tomato (64–84%) (Table 5). The sex ratio of adults varied among replications but was not affected by host plants (“plant effect”, *df* = 3, *p* = 0.15) nor by the resistance traits (“strain effect”, *df* = 3, *p* = 0.48) (Fig. S1, Table S2).

### Survivorship of *T. ni* is affected by the mechanisms of resistance and host plants

**Figure 2:**
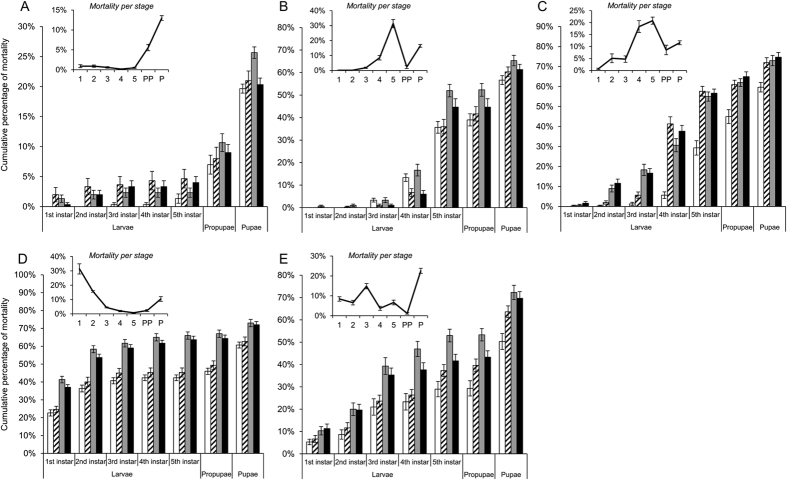
Cumulative percentage of mortality at each developmental stage for the susceptible (white), Cry1Ac (striped), Cry2Ab (grey) and Cry1Ac-Cry2Ab resistant strains (black) reared on artificial diet (**A**), cabbage (**B**), cotton (**C**), tobacco (**D**) or tomato (**E**). The inserts show the percentage of mortality at each stage. Values are presented as mean ± SE.

On artificial diet, 80.3% of the larvae from the susceptible strain completed pupation, but the pupation rate decreased to 39.3–49.7% on plants (“plant effect”, *df* = 4, *p* < 2.2 10^−16^) (Fig. 2, Fig. S1). The resistant strains had a pupation rate of 74.3–79.7% on artificial diet and of 25.3–39.7% when fed on the plant foliage. The effects of the host plants on survival of *T. ni* showed different larval stage specific patterns (“stage x plant”, *df* = 4, *p* < 2.2 10^−16^) (Fig. 2, Fig. S1). For *T. ni* on artificial diet, mortality mainly occurred during pre-pupation and at pupal stage (Fig. 2A). However, mortality predominantly occurred at 5^th^ instar and pupal stage on cabbage (Fig. 2B). *T. ni* larvae fed on cotton had high mortality at 4^th^ and 5^th^ larval instars and moderate mortality (about 10%) during prepupal and pupal stages (Fig. 2C). High mortality happened at 1^st^ and 2^nd^ larval instars on tobacco (Fig. 2D) and at 3^rd^ larval instar and pupal stage on tomato (Fig. 2E). The effect of different strains on mortality was also observed to be developmental stage-specific (“strain effect”, *df* = 3, *p* = 1.5 10^−03^) (Fig. S1), showing a generally higher mortality in GLEN-Cry2Ab-BCS and GLEN-Cry1Ac+Cry2Ab-BCS resistant strains as compared with the susceptible strain in nearly all instars on all plants (Fig. 2).

## Discussion

In this study, the fitness costs associated with two independent resistance mechanisms to two agriculturally important Bt toxins, individually and in combination, on four different crop plants were determined under the same genetic background in a lepidopteran pest, using the unique near-isogenic *T. ni* strains in the same genetic background of a laboratory *T. ni* strain. The laboratory strain has been maintained on artificial diet in laboratory and is well adapted to artificial diet, which may lead to their response to plant materials different from the *T. ni* from field populations. However, use of *T. ni* strains in the genetic background of the laboratory strain helps minimize the influence on insect performance by the unknown heterogeneous backgrounds of *T. ni* in the field populations that may have differential preference to different host plants. This experimental approach allowed examination of fitness costs on specific host plants associated with particular resistance mechanisms in an insect pest. Mechanisms of insect resistance to Bt toxins may vary from case to case, but are known not to be insect species-specific^29^,^32^. The Cry1Ac resistant *T. ni* individuals can survive on Cry1Ac-broccoli and Cry1Ac-cotton plants^25^,^30^ and the resistance is conferred by a mutation in the *ABCC2* gene locus region of the genome^28^. Such a high level of Cry1Ac resistance with genetic linkage to the *ABCC2* gene has also been identified in *H. virescens*^27^, *P. xylostella*^28^ and *B. mori*^33^. Therefore, the fitness costs determined in this study may represent the characteristics of fitness costs associated with the specific resistance mechanisms to Cry1Ac and to Cry2Ab in Lepidoptera.

**Figure 3:**
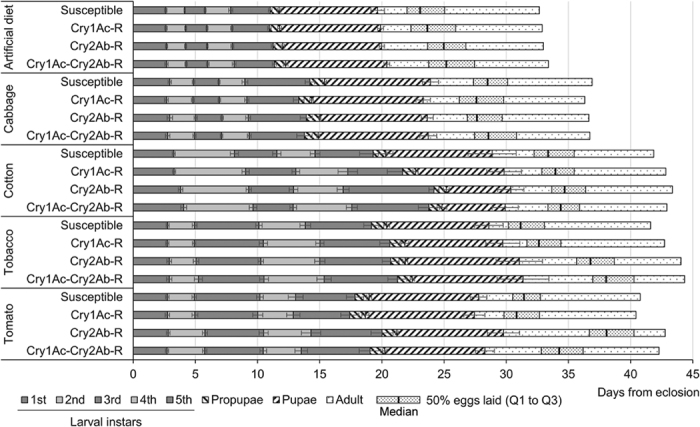
Mean individual total development time based on larval (dark and light grey) and pupal stage durations (hatched) (Table 4). Adult stage duration (dotted) was fixed at 13 days as adults were not reared until death in this study. The median, which is the number of days when 50% of total amount of eggs are laid, is indicated by a thick black line. A dotted box indicates the range of days around the median in which 50% of eggs are laid (between the 1^st^ and 3^rd^ quartiles).

Similar to many lepidopteran pests, *T. ni* is highly polyphagous. Although the mechanisms of Cry1Ac- and Cry2Ab-resistance in *T. ni* are associated with fitness costs, the resistant *T. ni* larvae can survive and complete their life cycle on their preferred primary host plant as well as on the secondary host plants cotton, tobacco and tomato, with their NRR and IRI values (*R*_*o*_ and *r*) far above the critical values (*R*_*o*_ > 1; *r* > 0) for population growth. The fitness of *T. ni* was always higher on artificial diet than on the host plants tested, irrespective of the *T. ni* strains, which is similar to the observations made in *P. xylostella*^8^ and *Pseudaletia sequax*^34^. Cabbage is a preferred host plant for *T. ni* and, as a crucifer, it produces glucosinolates as major anti-herbivory metabolites; whereas tobacco, tomato and cotton plants are known to produce alkaloids (*e.g.* nicotine), phenolics, protease inhibitors, and other insecticidal secondary metabolites^35^. Among the four host plants tested, cabbage was the one on which *T. ni* laid the largest number of eggs, developed the fastest, had the lowest mortality and the highest pupal weight, irrespective of the resistance traits. Cabbage appears to be the best-adapted host plant for *T. ni*, which is in agreement with the preference of *T. ni* on plants of the Brassicaceae family for host plant choice. These results are also consistent with the previous report that DiPel-resistant *T. ni* developed faster, survived better and weighed more when feeding on cucumber (family: Brassicaceae) plants than on tomato (family: Solanaceae) and pepper (family: Piperaceae) plants^14^. The results in this study showed that the *T. ni* strains all developed more slowly on cotton, tobacco and tomato foliage than on cabbage. The slower larval development on the secondary host plants resulted in the life cycle of *T. ni* to be 3.6 to 7.9 days longer and in asynchrony in adult emergence and egg laying than those feeding on cabbage (Fig. 3). Such a significant difference in developmental time to reach adult emergence between the primary and secondary host plants has also been observed in *S. frugiperda* on maize, cotton and soybean^21^, in *S. litura* on tobacco, sweet potato, cabbage and cow pea^22^ and in *T. ni* on cucumber, tomato and pepper plants^14^. The slower larval development of *T. ni* is associated with a lower pupal weight on cotton, tobacco and tomato as compared to feeding on cabbage. Interestingly, the patterns of mortality occurrence in *T. ni* at different developmental stages differed when *T. ni* were on different host plants. The difference observed on the four plants may reflect differential adaptation of *T. ni* larvae to these host plants. The results from this study and previous reports of similar studies in *T. ni*^14^, *H. armigera*^15^ and *P. xylostella*^11^,^13^ consistently show that the level of fitness costs is affected by the host plants on which the resistant insects feed on.

Mechanisms of insect resistance to Cry1Ac have been studied in several species of Lepidoptera^32^. Cry1A resistance may involve changes of proteases that affect the proteolytic processing of toxins^36^, but high levels of resistance usually involve changes of a midgut receptor for Cry toxins^32^. The Cry1Ac resistance in *T. ni* is associated with down-regulation of the *APN1* gene expression genetically linked to a mutation in the *ABCC2* gene locus region which acts in *trans*^28^,^37^. At present, genetic mechanisms for Cry2Ab resistance in *T. ni* is unknown, but it is known that the resistance to Cry1Ac and that to Cry2Ab are conferred by two genetically independent mechanisms^26^,^30^. The results from this study determined that the Cry1Ac resistance mechanism with a genetic linkage to the *ABCC2* locus is associated with a low fitness cost in *T. ni*. In comparison with the Cornell strain, the GLEN-Cry1Ac-BCS strain only shows a moderately extended larval development time, an increased mortality on cotton, and a slight decreased percentage of egg hatching. In contrary, the fitness costs associated with the Cry2Ab resistance mechanism were higher with significantly reduced fecundity and increased mortality. The GLEN-Cry1Ac+Cry2Ab-BCS strain is resistant to both Cry1Ac and Cry2Ab toxins, but no significant additive effect from the Cry1Ac and Cry2Ab resistance on the fitness costs was observed in this *T. ni* strain. Therefore, the fitness costs observed in the GLEN-Cry1Ac+Cry2Ab-BCS strain were not the result of addition of the costs associated with the two distinct resistance mechanisms. The effects of the different crop plants on the Bt resistance-associated fitness costs were determined in *T. ni* in this study, but the information could be extended to other lepidopterans which often have similar midgut physiology and may share the same Bt resistance mechanisms^32^.

The “high dose-refuge” strategy is a major tactic used to delay the development of insect resistance to Bt crops in the field^38^. This resistance management strategy relies on extensive mating between resistant insects from Bt crop fields and susceptible insects from non-Bt refuge zones^39^. The observations on adult emergence and egg laying of *T. ni* with a different Bt resistant trait and growing on different host plants showed both host plant-dependent and resistance trait-dependent asynchrony in adult emergence and egg laying dynamics (Fig. 3). Particularly important was the effect associated with Cry2Ab resistance in *T. ni* when fed on tobacco and tomato: the peak of egg laying was delayed 4–8 days, compared to the susceptible strain (Fig. 3). Given that *T. ni* females mostly mate within 3–5 days after emergence^40^, an asynchrony of adult emergence and egg laying of several days between the susceptible and resistant individuals on different plants per generation could potentially lead to significant phenological changes of the susceptible and resistant populations after multiple generations. This can be sufficient to affect the mating between resistant and susceptible insects and reduce the efficacy of the “high dose-refuge” strategy. This phenomenon might be amplified by the natural tendency of assortative mating between insects developing on the same plants and of transgenerational adaptation to the plant that can be at the basis of host plant shifts^41^,^42^. Altogether, the results from this study indicate that the relative fitness of Bt resistant insects compared to the susceptible insects depends on the specific resistance mechanisms and on host plants. Both insect resistance mechanisms and host plants available to the insects in the field are important factors affecting selection of resistance alleles in the field and therefore need be taken into account for development of resistance management strategies^20^,^43^.

## Materials and Methods

### Insect Strains

A highly inbred laboratory *T. ni* strain, the Cornell strain^30^, was used as the Bt susceptible control strain. A Cry1Ac resistant strain, GLEN-Cry1Ac-BCS^25^, a Cry2Ab resistant strain, GLEN-Cry2Ab-BCS^26^, and a strain resistant to both Cry1Ac and Cry2Ab, GLEN-Cry1Ac+Cry2Ab-BCS^30^, were used in this study to examine the fitness costs associated with the resistance traits on different crop plants. The Cry resistance traits in these three strains were originated from the greenhouse-derived GLEN-DiPel strain^31^ and the strains were all near-isogenic to the Cornell strain by introgression of the Cry resistance traits into the Cornell strain^25^,^26^. The *T. ni* strains were routinely maintained on artificial diet without exposure to Bt toxins^30^.

### Plants

Plants of cabbage variety Quisor (Stokes Seeds, Buffalo, NY), cotton variety Stoneville 474^30^, tobacco variety Wisconsin 38 (provided by Dr. Susheng Gan of Cornell University) and tomato variety Better Boy (Harris Seeds, Rochester, NY) were grown in formulated soil, the Cornell Mix, in 6 L plastic pots in the greenhouse at 27 ± 2 °C under a light and dark regime of 16:8 h^30^.

### Examination of Fitness Costs

Performance of *T. ni* larvae from the susceptible Cornell strain and three resistant strains was examined by rearing the larvae on artificial diet and on detached leaves of the cabbage, cotton, tobacco and tomato plants in 8.0 cm (diameter) ×6.5 cm (height) paper cups. A total of 300 neonates of each strain were placed in 30 cups (10 larvae per cup), provided with artificial diet or detached leaves of a test plant. Plant leaves were replaced daily or more frequently as necessary when the larvae reached 5^th^ instar. The cups were placed in an insect rearing chamber at 25 ± 1 °C, 50 ± 10% relative humidity, and 16:8 h photoperiod. Larval development and mortality were recorded every 12 h till completion of pupation. Pupae were collected daily and weighed. The sex of pupae was visually determined following the criteria described by Butt and Cantu^44^.

For examination of fecundity, one female moth and two male moths from the same cup were placed in a wire cage (12 cm in diameter and 11 cm in height) which was wrapped with wax paper for egg collection. The moths were provided with 10% sugar solution. For each treatment, 30 “1-female+2-males” cages (one “1-female+2-males” cage for each cup) were used for replications. The wax paper was replaced daily from each cage. Eggs on the wax paper were counted and hatching of the eggs was examined daily afterwards.

Two demographic parameters, net reproductive rate (*R*_*0*_) and intrinsic rate of increase (*r*), were calculated for each strain on each test plant using the formula described by Carey^45^. The net reproductive rate was calculated as *R*_*0*_ = N_n+1_/N_n_, where N_n_ is the population quantity of the parent generation (neonate number = 300) and N_n+1_ is that of the next generation. A *R*_*0*_ value of 1 indicates that females are having exactly enough offspring females to replace them in the next generation and *R*_*0*_ > 1 indicates that more daughters than mothers are present in the population. The intrinsic rate of increase was calculated as *r* = ln(*R*_*0*_)/((*x* * *l*_*x*_ * *m*_*x*_)/*R*_*0*_), where *x* is the females age in days, *l*_*x*_ the age-specific survival, *m*_*x*_ the age-specific fecundity and *R*_*0*_ the net reproductive rate. A strictly positive *r* value indicates that the population size is increasing while *r* < 0 is indicative of a collapsing population. The relative fitness (*w*) of the resistant strain was calculated as the ratio of *R*_*0*_ or *r* of the resistant strain to *R*_*0*_ or *r* of the susceptible strain.

### Statistical Analysis

The fitness parameters recorded from the experiments were analyzed using a generalized linear model (GLM) to measure the effect of different factors (*i.e.* strain, host plant) on the fitness of *T. ni* and the interactions between the factors. In all data analyses, the individual cups were considered as independent units to be analyzed, using the mean of data from 10 larvae in each cup or from a “1-female+2-males” for each mating group. Normality of the data was verified by using a Shapiro-Wilk test. In order to determine the most suitable distribution model for the GLM, a one-sample Kolmogorov-Smirnov test against the corresponding model was used. A binomial error family was used for traits expressed as percentages (hatching, emergence and survival) while a Poisson error family was used for all other traits. When a significant effect was found, an ANOVA followed by multiple pairwise comparisons of means (Tukey’s HSD test) was performed in order to determine the effect of each strain and each host plant on the fitness parameters analyzed. Pairwise comparisons of egg laying kinetics were performed with a two-sample two-sided Kolmogorov-Smirnov test. All statistical analyses were performed using the software R 3.0.2^46^.

## Additional Information

**How to cite this article**: Wang, R. *et al.* Effect of crop plants on fitness costs associated with resistance to *Bacillus thuringiensis* toxins Cry1Ac and Cry2Ab in cabbage loopers. *Sci. Rep.* **6**, 20959; doi: 10.1038/srep20959 (2016).

## Supplementary Material

Supplementary Information

## Figures and Tables

**Table 1:** Net reproductive rate and relative fitness of four *T. ni* strains on artificial diet and plant leaves.

Host plant	Parameter	*T. ni* strain			
		Susc.^a^	1Ac-R^b^	2Ab-R^c^	1Ac2Ab-R^d^
Artificial diet	NRR (*R*_0_)^e^	347.9	277.7	280.0	252.3
	Fitness (*w*)^f^	1.00	0.80	0.80	0.72
Cabbage	NRR (*R*_0_)^e^	111.2	96.2	98.7	86.7
	Fitness (*w*)^f^	1.00	0.86	0.89	0.78
Cotton	NRR (*R*_0_)^e^	90.8	48.7	39.7	33.1
	Fitness (*w*)^f^	1.00	0.54	0.44	0.36
Tobacco	NRR (*R*_0_)^e^	98.2	76.7	50.7	45.4
	Fitness (*w*)^f^	1.00	0.78	0.52	0.46
Tomato	NRR (*R*_0_)^e^	123.2	61.2	31.0	40.4
	Fitness (*w*)^f^	1.00	0.50	0.25	0.33

^a^Cornell Bt-susceptible strain.

^b^GLEN-Cry1Ac-BCS strain.

^c^GLEN-Cry2Ab-BCS strain.

^d^GLEN-Cry1Ac+Cry2Ab-BCS strain.

^e^Net reproductive rate (NRR): *R*_*0*_ = N_n+1_/N_n_. N_n_ is the population quantity (neonate number) of the parent generation and N_n+1_ is that of the next generation.

^f^Relative fitness (*w*) = *R*_*0*_(resistant strain)/*R*_*0*_(susceptible strain).

**Table 2:** Intrinsic rate of increase and relative fitness of four *T. ni* strains on artificial diet and plant leaves.

Host plant	Parameter	*T. ni* strains			
		Susc.^a^	1Ac-R^b^	2Ab-R^c^	1Ac2Ab-R^d^
Artificial diet	IRI (*r*)^e^	0.297	0.283	0.282	0.271
	Fitness (*w*)^f^	1.00	0.95	0.95	0.91
Cabbage	IRI (*r*)^e^	0.197	0.195	0.194	0.188
	Fitness (*w*)^f^	1.00	0.99	0.98	0.95
Cotton	IRI (*r*)^e^	0.158	0.130	0.121	0.117
	Fitness (*w*)^f^	1.00	0.82	0.77	0.74
Tobacco	IRI (*r*)^e^	0.160	0.146	0.128	0.122
	Fitness (*w*)^f^	1.00	0.91	0.80	0.76
Tomato	IRI (*r*)^e^	0.173	0.150	0.116	0.131
	Fitness (*w*)^f^	1.00	0.86	0.67	0.76

^a^Cornell Bt-susceptible strain.

^b^GLEN-Cry1Ac-BCS strain.

^c^GLEN-Cry2Ab-BCS strain.

^d^GLEN-Cry1Ac+Cry2Ab-BCS strain.

^e^Intrinsic rate of increase (IRI): *r* = ln(*R*_*0*_)/((*x* * *l*_*x*_ * *m*_*x*_)/*R*_*0*_). *x* is the females age in days, *l*_*x*_ is the age-specific survival, *m*_*x*_ is the age-specific fecundity and *R*_*0*_ is the net reproductive rate.

^f^Relative fitness (*w*) = *r*(resistant strain)/*r*(susceptible strain).

**Table 3:** Egg laying and egg hatchability of four *T. ni* strains on artificial diet and plant leaves.

	Strain	Artificial diet			Cabbage			Cotton			Tobacco			Tomato		
Total no. of eggs laid per moth	Susc.^a^	882.5 ± 151.4	a	A	702.4 ± 182.2	a	B	572.9 ± 150.6	a	B	696.5 ± 155.4	a	B	543.7 ± 156.3	a	B
	1Ac-R^b^	834.5 ± 127.7	a	A	649.2 ± 154.3	a	BC	495.4 ± 124.1	a	B	603.7 ± 184.7	a	B	432.6 ± 100.6	a	C
	2Ab-R^c^	820.9 ± 138.2	a	A	680.3 ± 183.3	a	AB	510.9 ± 123.6	a	C	548.5 ± 98.5	a	BC	378.3 ± 78.7	a	C
	1Ac-2Ab-R^d^	812.9 ± 154.4	a	A	675.3 ± 150.7	a	AB	476.3 ± 105.6	a	C	518.7 ± 106.1	a	BC	406.1 ± 111.3	a	C
Egg hatching (%)	Susc.^a^	85.7 ± 1.8	a	A	82.0 ± 4.5	a	A	83.4 ± 3.3	a	A	83.6 ± 5.2	a	A	80.9 ± 5.0	a	A
	1Ac-R^b^	83.2 ± 1.7	ab	A	77.0 ± 5.3	a	B	77.6 ± 4.2	b	B	79.9 ± 5.7	a	AB	75.8 ± 5.2	a	B
	2Ab-R^c^	81.2 ± 3.3	b	A	77.8 ± 4.4	a	AB	72.9 ± 5.1	b	B	79.2 ± 4.2	a	A	76.6 ± 6.4	a	AB
	1Ac-2Ab-R^d^	83.9 ± 2.2	ab	A	75.8 ± 5.6	b	B	74.5 ± 4.3	b	B	77.2 ± 4.5	b	B	74.7 ± 7.5	b	B

^*^Significant differences between different *T. ni* strains on the same plants are indicated by different letters in lower case in the same column. Significant differences for the same strain between different plants are indicated by different letters in upper case in the same row. Values are indicated as mean ± SD.

^a^Cornell Bt-susceptible strain.

^b^GLEN-Cry1Ac-BCS strain.

^c^GLEN-Cry2Ab-BCS strain.

^d^GLEN-Cry1Ac+Cry2Ab-BCS strain.

**Table 4:** Larval and pupal development of four *T. ni* strains on artificial diet and plant leaves.

	Strain	Artificial diet			Cabbage			Cotton			Tobacco			Tomato		
Larval stage duration (days)	Susc.^a^	11.02 ± 0.28	a	A	14.22 ± 0.39	a	B	19.30 ± 1.39	a	C	19.16 ± 0.89	a	C	17.84 ± 0.99	a	C
	1Ac-R^b^	10.95 ± 0.24	a	A	13.31 ± 0.50	b	B	21.67 ± 1.31	b	C	20.64 ± 0.99	b	D	17.42 ± 0.88	a	E
	2Ab-R^c^	11.25 ± 0.24	a	A	13.92 ± 0.50	a	B	24.18 ± 0.72	c	C	20.70 ± 1.31	b	D	20.01 ± 1.14	b	D
	1Ac-2Ab-R^d^	11.36 ± 0.37	a	A	13.77 ± 0.68	a	B	23.78 ± 0.72	c	C	21.25 ± 1.31	b	C	19.05 ± 1.21	b	C
Pupal stage duration (days)	Susc.^a^	7.92 ± 0.54	a	A	8.52 ± 0.65	a	A	8.57 ± 1.93	a	A	8.22 ± 1.15	ab	A	8.80 ± 0.67	a	A
	1Ac-R^b^	8.12 ± 0.23	a	AB	8.97 ± 0.58	a	A	7.12 ± 1.39	a	B	7.97 ± 1.37	a	BC	8.77 ± 0.85	a	AC
	2Ab-R^c^	7.96 ± 0.20	a	A	8.63 ± 0.48	a	AC	5.19 ± 1.05	b	B	9.17 ± 1.87	b	C	8.58 ± 1.30	a	AC
	1Ac-2Ab-R^d^	8.13 ± 0.23	a	A	8.85 ± 0.67	a	A	5.08 ± 1.04	b	B	8.90 ± 2.08	ab	A	8.05 ± 0.76	a	A

^*^Significant differences between different *T. ni* strains on the same plants are indicated by different letters in lower case in the same column. Significant differences for the same strain between different plants are indicated by different letters in upper case in the same row. Values are indicated as mean ± SD.

^a^Cornell Bt-susceptible strain.

^b^GLEN-Cry1Ac-BCS strain.

^c^GLEN-Cry2Ab-BCS strain.

^d^GLEN-Cry1Ac+Cry2Ab-BCS strain.

**Table 5:** Pupal weight and percentage of emergence of four *T. ni* strains on artificial diet and plant leaves.

	Strain	Artificial diet			Cabbage			Cotton			Tobacco			Tomato		
Pupal weight (mg)	Susc.^a^	205.2 ± 24.4	a	AB	222.6 ± 54.0	a	A	186.6 ± 55.6	a	B	199.0 ± 24.1	a	AB	137.1 ± 35.7	a	C
	1Ac-R^b^	212.6 ± 19.2	a	A	218.8 ± 42.7	a	A	175.9 ± 51.5	a	B	188.5 ± 32.5	a	B	140.2 ± 47.0	a	C
	2Ab-R^c^	193.3 ± 22.9	a	AB	216.1 ± 45.3	a	A	173.0 ± 42.5	a	B	184.2 ± 40.4	a	B	106.7 ± 50.5	b	C
	1Ac-2Ab-R^d^	211.2 ± 25.7	a	AC	231.9 ± 50.2	a	A	185.6 ± 42.8	a	B	190.4 ± 47.7	a	C	146.5 ± 40.2	a	D
Emergence of healthy adults (%)	Susc.^a^	86.16 ± 14.6	a	A	71.3 ± 11.8	a	A	74.0 ± 14.7	a	A	73.5 ± 16.2	a	A	72.0 ± 19.6	a	A
	1Ac-R^b^	85.77 ± 10.1	a	A	69.5 ± 15.3	a	AB	71.7 ± 22.3	a	AB	74.9 ± 19.8	a	AB	62.3 ± 20.6	a	B
	2Ab-R^c^	82.74 ± 13.7	a	A	74.6 ± 14.0	a	AB	72.3 ± 20.5	a	AB	75.2 ± 31.7	a	AB	62.7 ± 23.6	a	B
	1Ac-2Ab-R^d^	87.32 ± 11.1	a	A	71.6 ± 20.6	a	AB	75.7 ± 20.1	a	A	75.3 ± 29.5	a	A	56.0 ± 20.0	a	B

^*^Significant differences between different *T. ni* strains on the same plants are indicated by different letters in lower case in the same column. Significant differences for the same strain between different plants are indicated by different letters in upper case in the same row. Values are indicated as mean ± SD.

^a^Cornell Bt-susceptible strain .

^b^GLEN-Cry1Ac-BCS strain.

^c^GLEN-Cry2Ab-BCS strain.

^d^GLEN-Cry1Ac+Cry2Ab-BCS strain.
