# Supplementary material for: Effect of crop plants on fitness costs associated with resistance to Bacillus thuringiensis toxins Cry1Ac and Cry2Ab in cabbage loopers
Source: Sci Rep. 2016 Feb 12;6:20959. doi: 10.1038/srep20959 (PMC4751490; doi:10.1038/srep20959)
Supplement: Supplementary Information [file srep20959-s1.pdf]

SUPPLEMENTARY FILES

Effect of crop plants on fitness costs associated with resistance to *Bacillus thuringiensis* toxins Cry1Ac and Cry2Ab in cabbage loopers

Ran Wang[a,b,1,2], Guillaume Tetreau[a,1,3], Ping Wang[a,*]

[a]Department of Entomology, Cornell University, New York State Agricultural Experiment Station, Geneva, NY 14456, USA

[b]Department of Entomology, Nanjing Agricultural University, Nanjing 210095, China.

[1]R.W. and G.T. contributed equally to this work.

[2]Present address: The Institute of Plant and Environment Protection, Beijing Academy of Agriculture and Forestry Sciences, Beijing 100097, China.

[3]Present address: Univ. Perpignan Via Domitia, IHPE UMR 5244, CNRS, IFREMER, Univ. Montpellier, F-66860 Perpignan, France.

*To whom correspondence should be addressed. E-mail: pingwang@cornell.edu.

Figure S1. Summary of analysis of deviance tables of all GLM performed on all life parameters. Factors are indicated as they were added sequentially to the model, from first to last. A binomial error family was used for traits expressed as percentages (hatching, emergence, and survival) while a Poisson error family was used for all other traits. Chi-square *p* values indicate the level of significance of the various factors in the model as compared to the Null model (no effect included). '*, $p < 0.05$; '**', $p < 0.01$; '***', $p < 0.001$.

DEVELOPMENT (FROM EGG TO ADULT) – Poisson error

	Df	Deviance	Resid. Df	Resid. Dev	Pr(>Chi)	
NULL			4189	5788.0		
Devel.Stage	6	4331.6	4183	1456.3	< 2.2e-16	***
Plant	4	368.1	4179	1088.2	< 2.2e-16	***
Strain	3	5.5	4176	1082.7	0.139825	
Devel.Stage:Plant	24	734.0	4152	348.7	< 2.2e-16	***
Devel.Stage:Strain	18	37.1	4134	311.6	0.005081	**
Plant:Strain	12	4.4	4122	307.2	0.975177	
Devel.Stage:Plant:Strain	72	80.8	4050	226.4	0.224394	

EGG HATCHING – Binomial error

	Df	Deviance	Resid. Df	Resid. Dev	Pr(>Chi)	
NULL			421	1.8098		
Strain	3	0.26378	418	1.5461	< 2.2e-16	***
Plant	4	0.36486	414	1.1812	< 2.2e-16	***
Strain:Plant	12	0.08574	402	1.0955	0.001573	**

EGG LAYING KINETICS – Poisson error

	Df	Deviance	Resid. Df	Resid. Dev	Pr(>Chi)	
NULL			6783	589239		
Day	1	130739	6782	458500	< 2.2e-16	***
Plant	4	13953	6778	444547	< 2.2e-16	***
Strain	3	1341	6775	443206	< 2.2e-16	***
Plant:Strain	11	1243	6764	441963	< 2.2e-16	***
Day:Plant	4	276	6760	441687	< 2.2e-16	***
Day:Strain	3	9375	6757	432312	< 2.2e-16	***
Day:Plant:Strain	11	9481	6746	422831	< 2.2e-16	***

EMERGENCE OF HEALTHY ADULTS – Binomial error

	Df	Deviance	Resid. Df	Resid. Dev	Pr(>Chi)	
NULL			599	155.85		
Plant	4	16.3321	595	139.52	0.002604	**
Strain	3	0.3170	592	139.20	0.956802	
Plant:Strain	12	2.0559	580	137.14	0.999315	

FECUNDITY (TOTAL NUMBER OF EGGS PER FEMALE) - Poisson error

	Df	Deviance	Resid. Df	Resid. Dev	Pr(>Chi)	
NULL			421	29247		
Plant	4	14091.0	417	15156	< 2.2e-16	***
Strain	3	978.1	414	14178	< 2.2e-16	***
Plant:Strain	12	590.4	402	13587	< 2.2e-16	***

LARVAL STAGE DURATION - Poisson error

	Df	Deviance	Resid. Df	Resid. Dev	Pr(>Chi)	
NULL			599	679.07		
Plant	4	622.12	595	56.95	< 2.2e-16	***
Strain	3	17.68	592	39.27	0.0005113	***
Plant:Strain	12	14.56	580	24.71	0.2661775	

LAYING DAYS (TOTAL NUMBER) - Poisson error

	Df	Deviance	Resid. Df	Resid. Dev	Pr(>Chi)	
NULL			421	283.80		
Plant	4	69.985	417	213.82	2.287e-14	***
Strain	3	1.811	414	212.01	0.6125	
Plant:Strain	12	6.116	402	205.89	0.9101	

MORTALITY (DEVELOPMENTAL STAGE-SPECIFIC)- Binomial error

	Df	Deviance	Resid. Df	Resid. Dev	Pr(>Chi)	
NULL			2999	731.24		
Stage	4	28.896	2995	702.34	8.209e-06	***
Strain	3	15.350	2992	686.99	0.001541	**
Plant	4	107.136	2988	579.85	< 2.2e-16	***
Strain:Plant	12	5.056	2976	574.80	0.956087	
Stage:Plant	16	231.952	2960	342.85	< 2.2e-16	***
Stage:Strain	12	3.461	2948	339.39	0.991319	
Stage:Strain:Plant	48	26.006	2900	313.38	0.996018	

PUPAL WEIGHT - Poisson error

	Df	Deviance	Resid. Df	Resid. Dev	Pr(>Chi)
NULL			1799	28.931	
Plant	4	9.3607	1795	19.570	0.05269
Strain	3	0.4812	1792	19.089	0.92301
Gender	1	0.0396	1791	19.050	0.84233
Plant:Strain	12	0.4731	1779	18.576	1.00000
Plant:Gender	4	0.1819	1775	18.395	0.99611
Strain:Gender	3	0.0157	1772	18.379	0.99948
Plant:Strain:Gender	12	0.1106	1760	18.268	1.00000

PUPAL STAGE DURATION – Poisson error

	Df	Deviance	Resid. Df	Resid. Dev	Pr(>Chi)	
NULL			594	187.377		
Plant	4	52.646	590	134.730	1.01e-10	***
Strain	3	4.297	587	130.433	0.2311	
Plant:Strain	12	39.345	575	91.088	9.23e-05	***

SEX RATIO – Poisson error

	Df	Deviance	Resid. Df	Resid. Dev	Pr(>Chi)
NULL			593	172.56	
Plant	3	5.3841	590	167.18	0.1457
Strain	3	2.4686	587	164.71	0.4810
Plant:Strain	9	2.6523	578	162.06	0.9765

Table S2. Sex ratio in each strain and each plant. Values are indicated as mean ± SD. The host plant (GLM, *df* = 3, *p* = 0.15), the strain (GLM, *df* = 3, *p* = 0.48) and their interaction (GLM, *df* = 9, *p* = 0.98) had no significant effect on sex ratio.

	Susceptible	Cry1Ac-R	Cry2Ab-R	Cry1Ac-Cry2Ab-R	Mean
Artificial diet	59.0 ± 17.6%	51.1 ± 16.5%	55.4 ± 19.9 %	47.0 ± 16.0 %	53.1 ± 17.5%
Cabbage	44.1 ± 15.6%	49.4 ± 22.8%	55.9 ± 24.7%	41.4 ± 24.8%	47.7 ± 22.0%
Cotton	46.0 ± 20.0%	45.1 ± 18.0%	39.2 ± 25.4%	36.9 ± 30.6%	41.8 ± 23.5%
Tobacco	40.5 ± 17.3%	44.1 ± 24.6%	37.1 ± 26.5%	37.6 ± 21.5%	39.8 ± 22.5%
Tomato	59.5 ± 23.8%	53.3 ± 24.9%	40.3 ± 32.3%	44.8 ± 25.6%	49.5 ± 26.7%
Mean	49.8 ± 18.9%	48.6 ± 21.4%	45.6 ± 25.8%	41.5 ± 23.7%	
